# Mitochondrial Respiration after One Session of Calf Raise Exercise in Patients with Peripheral Vascular Disease and Healthy Older Adults

**DOI:** 10.1371/journal.pone.0165038

**Published:** 2016-10-19

**Authors:** Michel van Schaardenburgh, Martin Wohlwend, Øivind Rognmo, Erney J. R. Mattsson

**Affiliations:** 1 Department of Circulation and Medical Imaging, Faculty of Medicine, Norwegian University of Science and Technology, Trondheim, Norway; 2 Department of Vascular Surgery, St. Olavs Hospital, Trondheim, Norway; Victoria University, AUSTRALIA

## Abstract

**Purpose:**

Mitochondria are essential for energy production in the muscle cell and for this they are dependent upon a sufficient supply of oxygen by the circulation. Exercise training has shown to be a potent stimulus for physiological adaptations and mitochondria play a central role. Whether changes in mitochondrial respiration are seen after exercise in patients with a reduced circulation is unknown. The aim of the study was to evaluate the time course and whether one session of calf raise exercise stimulates mitochondrial respiration in the calf muscle of patients with peripheral vascular disease.

**Methods:**

One group of patients with peripheral vascular disease (n = 11) and one group of healthy older adults (n = 11) were included. Patients performed one session of continuous calf raises followed by 5 extra repetitions after initiation of pain. Healthy older adults performed 100 continuous calf raises. Gastrocnemius muscle biopsies were collected at baseline and 15 minutes, one hour, three hours and 24 hours after one session of calf raise exercise. A multi substrate (octanoylcarnitine, malate, adp, glutamate, succinate, FCCP, rotenone) approach was used to analyze mitochondrial respiration in permeabilized fibers. Mixed-linear model for repeated measures was used for statistical analyses.

**Results:**

Patients with peripheral vascular disease have a lower baseline respiration supported by complex I and they increase respiration supported by complex II at one hour post-exercise. Healthy older adults increase respiration supported by electron transfer flavoprotein and complex I at one hour and 24 hours post-exercise.

**Conclusion:**

Our results indicate a shift towards mitochondrial respiration supported by complex II as being a pathophysiological component of peripheral vascular disease. Furthermore exercise stimulates mitochondrial respiration already after one session of calf raise exercise in patients with peripheral vascular disease and healthy older adults.

**Trial Registration:**

ClinicalTrials.gov NCT01842412

## Introduction

Mitochondria are known as the power plant of the muscle cell and are essential for energy production to maintain normal physiology and health. Energy is produced through the biological process of oxidative phosphorylation that occurs on the inner membrane of mitochondria. Of importance for oxidative phosphorylation is a sufficient supply of oxygen and an intact electron transfer system. The electron transfer system consists mainly of 4 protein complexes that transfer electrons over to oxygen [1]. This process is coupled to phosphorylation at a fifth complex where the energy molecule ATP is formed [2]. Oxidative phosphorylation can be assessed by mitochondrial respiration. Patients with Peripheral Vascular Disease (PVD) have a reduced blood flow to their lower extremity and thereby oxygen supply to the muscles is insufficient when increased energy is required. Furthermore, previous studies demonstrate reduced activities of electron transfer system complex I, complex III and complex IV in mitochondria of patients with PVD [3–5]. With age complex IV activity declines in sedentary humans and life long physical activity seems to preserve complex IV activity [6,7]. Exercise is known to stimulate pathways that induce mitochondrial adaptations [8]. One session of exercise stimulates mRNA expression of complex IV and is later followed by an increase of complex IV proteins in older adults [7,9].

Short periods of ischemia and reperfusion (ischemic preconditioning) preserve mitochondrial respiration in rodent skeletal muscle and the human heart [10,11]. Short sessions of exercise training continued into ischemia, may therefore preserve or ideally improve mitochondrial respiration in the calf muscle of patients with PVD.

To our best knowledge it is unknown whether exercise results in acute changes in mitochondrial respiration in patients with PVD. The aim of the study was to evaluate whether one session of calf raise exercise stimulates mitochondrial respiration in calf muscles of patients with PVD and healthy older adults as well as the time course for this potential effect to occur.

## Materials and Methods

### Participants

Patients with PVD (n = 11) were recruited between April 2013 and January 2014 at the department of vascular surgery, St Olavs University Hospital, Trondheim, Norway. Healthy older adults (n = 11) were recruited from the general population. All experimental protocols and procedures were approved by the regional committee of medical and health research ethics, central Norway (nr 2013/395) and conformed to the Declaration of Helsinki. Written informed consent was obtained from all participants. The study was registered in ClinicalTrials.gov (ID: NCT01842412).

### Eligibility assessment

Patients diagnosed with PVD, because of leg symptoms and an ankle-brachial index between 0.4 and 0.9 were included. Patients diagnosed with critical limb ischemia, vascular interventions in the last 3 months or having an ankle brachial index over 0.9 or under 0.4 were excluded. Healthy older adults without leg symptoms and an ankle brachial index over 0.9 were included. Exclusion criteria for both groups were usage of anticoagulants, diabetes mellitus, active cancer, renal- or liver disease.

### Intervention

Participants came to the hospital for skeletal muscle biopsy collection on two consecutive days. Participants were instructed not to perform exercise during the testing period. Smoking was not allowed before and during testing. Patients were fasted for 3 hours. On the first day biopsies were collected at baseline and at 15 minutes, 1 hour and 3 hours post-exercise. Patients with PVD were standing in front of a wall, which was used for support of the balance. The body was lifted with the calf musculature to the maximal height that the subject could achieve. This was repeated until pain was felt in the calf musculature. Following the initiation of pain the subject performed five extra repetitions. The healthy older adults performed 100 calf raises. On the first day participants maintained bed rest between biopsy collections. They left the research unit after the fourth biopsy and came back the next day for collection of the fifth muscle biopsy 24 hours post-exercise.

### Skeletal muscle biopsy

Biopsies were collected from the lateral part of the gastrocnemius muscle. In the patients with PVD biopsies were taken from the symptomatic leg and in the healthy older adults from the left leg. A micro biopsy technique was conducted to obtain muscle tissue [12]. Briefly, the sampling site was shaved. Five places were marked on the skin directly over the lateral part of the gastrocnemius muscle. The skin was then sterilized with chlorhexidine 5% and locally anesthetized by subcutaneous injection of Xylocain with adrenalin (Astra Zeneca, Oslo, Norway). The local anesthetic was strictly injected under the skin, to avoid influence of the muscle mitochondria. A 14 gauge insertion cannula (BioPince, Medical device technologies Inc., Gainesville, Florida USA) punctured the skin perpendicular to the muscle until the fascia was pierced. A sterile 16 gauge biopsy needle was introduced through the cannula and muscle biopsy samples were obtained from the gastrocnemius muscle.

### Permeabilized skeletal muscle fiber preparation

The muscle tissue was immediately transferred into ice-cold biopsy preservation solution (BIOPS) containing 10 mM Ca-EGTA buffer, 0.1 uM free calcium, 20mM imidazole, 20mM taurine, 50mM 2-(N-morpholino) ethane-sulfonic acid hydrate, 0.5mM dithiothreitol, 6.56 mM MgCl_2_, 5.77 mM ATP, 15 mM phosphocreatine (pH 7.1) [13]. A sample of the muscle tissue was transferred into a small petri dish filled with BIOPS and placed on an ice-cold metal plate. Muscle samples were then gently dissected using forceps and fibers were chemically permeabilized via incubation in 2 ml of BIOPS containing saponin (50 μg/ml) for 30 minutes. The objective was to permeabilize the extracellular membranes of the muscle fibers leaving intracellular membranes of the mitochondria intact. The muscle fibers were then washed for 10 min at 4 degrees Celsius in a mitochondrial respiration medium (MiR05) containing 110 mM sucrose, 60 mM K^+^-lactobionate, 0.5mM EGTA, 3mM MgCL_2_, 20 mM taurine, 10 mM KH_2_PO_4_, 20 mM HEPES and 1g/l bovine serum albumin (pH 7.1). The wet weight of muscle fibers (1-3mg) was measured on a microbalance (Sartorius ME235P-SD; Sartorius AG, Goettingen, Germany) immediately before assessment of mitochondrial respiration.

### Mitochondrial respiration measurements

The muscle fibers were transferred into a 2ml glass chamber containing MiR05 for high-resolution respirometry measurements (Oxygraph-2k; Oroboros Instruments, Innsbruck, Austria). The oxygen concentration and oxygen consumption were continuously recorded in the chamber. Oxygen consumption per second, per milligrams of wet weight of muscle fibers will be further addressed as being mitochondrial respiration (pmol O_2_ / s / mg wet weight of muscle fibers). Measurements were performed at 37°C All experiments were carried out in hyper-oxygenated chamber to prevent any potential oxygen diffusion limitation.

#### Respiratory Titration Protocol

The Substrate, Uncoupler and Inhibitor Titration (SUIT) protocol was used to examine different branches of the electron transfer system as previously described [13,14]. This was achieved by adding inductive or blocking substrates to the chamber. All respirometric analyses were made in duplicates and all titrations were added in series as presented. LEAK (_L_) state; electron transferring-flavoprotein (ETF) linked transfer of electrons in absence of ADP was induced with the addition of octanoyl carnitine (0.2 mM) and malate (2 mM). The LEAK state represents the resting mitochondrial respiration of an unaltered and intact electron transport system free of ADP. Malate was added because the ETF linked transfer of electrons requires the metabolism of acetyl-CoA to facilitate convergent electron flow into the Q-junction from both complex I (CI) and ETF (ETF+CI)_L_. The rate-limiting metabolic branch is electron transfer through ETF and not the contribution of electron flow through complex I. OXPHOS (_P_) state; maximal electron flow through ETF was determined following the addition of ADP (2,5 mM). Malate-octanoylcarnitine-ADP-stimulated respiration is representative of electron capacity through ETF (ETF+CI)_P_. Mitochondrial respiration specific to complex I (CI+ETF)_P_, was induced following the addition of glutamate (10 mM). Respiration supported by complex I and complex II (CI+CII+ETF)_P_, was then induced with the addition of succinate (10 mM) and ADP (5mM). (CI+CII+ETF)_P_ demonstrates a naturally intact electron transport system’s capacity to catalyze a sequential set of redox reactions that are partially coupled to the production of ATP at complex V [13,15]. As an internal control for compromised integrity of the mitochondrial preparation, the mitochondrial outer membrane was assessed with the addition of cytochrome *c* (10 uM). Data was excluded when an increase of 20% in respiration was found. Electron transfer system (_E_) state; phosphorylative restraint of electron transport was assessed by uncoupling complex V from the electron transport system with the titration of the proton ionophore, carbonyl cyanide *p*-(trifluoromethoxy) phenylhydrazone (FCCP: 0.5 M stepwise titration to optimum concentrations ranging from 1.5 to 3 M), thereby reaching electron transfer system capacity (CI+CII+ETF)_E_.Finally, rotenone (0.5 M), malonic acid (5mM) and antimycin A (2.5 M) were added, in sequence, to terminate respiration by inhibiting complex I, complex II and complex III respectively. With complex I inhibited, electron flow specific to complex II (CII) _E_ can be measured.

#### Mitochondrial function

The oxphos coupling efficiency, the excess electron transfer system-phosphorylation factor capacity and complex II control factor were calculated as measures of mitochondrial quality and control. Oxphos coupling efficiency, calculated with the formula (1- (ETF+CI)_L_ / (ETF+CI)_P_), reflects the coupling of respiration supported by electron transfer flavor protein (ETF) with octanoylcarnitin and malate as substrates before (ETF+CI)_L_ and after addition of ADP (ETF+CI)_P_. The excess electron transfer system-phosphorylation factor capacity (1 –(CI+CII+ETF)_P_/(CI+CII+ETF)_E_) is an expression of the relative limitation of oxphos capacity (CI+CII+ETF)_P_ by the electron transport system capacity (CI+CII+ETF)_E_ of the phosphorylation system. The complex II control factor (1-(CI+ETF)_P_/(CI+CII+ETF_P_)) reflects the fractional change of mitochondrial respiration when succinate (CI+CII+ETF)_P_ is added to respiration supported by complex I (CI+ETF)_P_.

#### Citrate synthase activity

Citrate synthase activity was assayed in homogenates of the permeabilized fibers used in the respiration measurements [14]. The content of the chambers was removed after each respiration experiment and washed with 0.2 ml MiR05 for 10 minutes at 4 degrees. The fluid was frozen at—80 degrees. Citrate synthase activity was measured later with a spectro-photometer at 412 nm and 25°C (Citrate Synthase Assay Kit, Sigma-Aldrich), according to the manufacturer’s instructions.

### Statistical analyses

The statistical power was calculated based on a paired t-test with correlated means. Data from a previous study [16] was used. The mean of group 1 was 123 with a standard deviation of 18. The mean of group 2 was 101 with a standard deviation of 24. A correlation of the means of 0.65, one-sided significance level of 0.05 and a power of the test of 80% indicated a need of 8 participants to be included in each group. This was increased to 10 participants, because of intention-to-treat analysis. Baseline characteristics and continuous variables are depicted as mean and standard error of the mean. Assessment of mitochondrial respiration and citrate synthase activity was performed blinded. Mitochondrial respiration between groups at baseline and within groups post-exercise was analyzed using mixed linear models for repeated measures. Missing data was assumed to be missing at random. A sensitivity analysis excluding patients with missing values was performed (complete-case analysis) to determine whether this might affect the main results. Furthermore, 3 post hoc sensitivity analyses (the lowest value, highest value measured and last observation carried forward) were performed. Participants were not age- or sex-matched so that each participant served as his or her own control. A p-value < 0.05 was considered significant. All analyses were performed using the statistical software STATA 13^th^ edition (StataCorp LP, Texas, USA).

## Results

**Fig 1:**
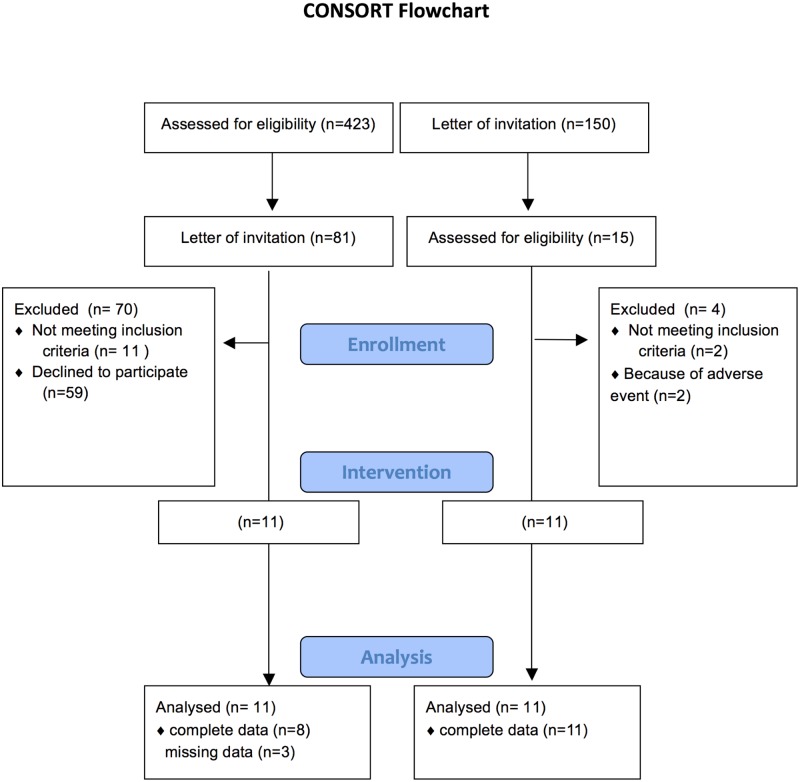
CONSORT flowchart.

A total of 11 patients with PVD and 11 older adults were included (Fig 1). Because of a technical failure data is missing at random from 3 participants in the patients group. 2 older adults experienced adverse events (bleeding after the first biopsy) and data is therefore not used for statistical analyses.

### Baseline characteristics

All baseline characteristics are presented in Table 1. There were differences in sexes; the patient group consisted of 9 male and 2 female participants. The group of healthy older adults consisted of 2 male and 9 female participants. Ankle brachial index was 0.65 ± 0.19 in the patient group versus 1.10 ± 0.08 in the healthy older adults.

**Table 1:** Baseline characteristics.

	patients with PVD (n = 11)	healthy older adults (n = 11)	p value
**Male/female gender**	9/2	2/9	
**Age (years)**	65 ± 6	73 ± 2	p = 0.002
**Height**	178 ± 6.3	166.5 ± 6.3	p = 0.000
**Weight**	83.8 ± 11.9	67 ± 12.7	p = 0.002
**Systolic blood pressure (mmHg)**	140 ± 8	129 ± 12	p = 0.01
**Diastolic blood pressure (mmHg)**	76 ± 9	74 ± 12	p = 0.33
**Ankle brachial index**	0.65 ± 0.19	1.10 ± 0.08	p = 0.000
**Body mass index**	26.3 ± 3	24 ± 3	p = 0.043
**Maximal walking distance (meters)**	254 ± 26	n.a.	
**Calf raises performed median (range)**	65 (38–210)	100	
**Currently smoking**	2	0	

Data are expressed with mean ± standard deviation of the mean. The p-values have been generated by the use of an independent samples t-test.

### Mitochondrial function at baseline

**Fig 2:**
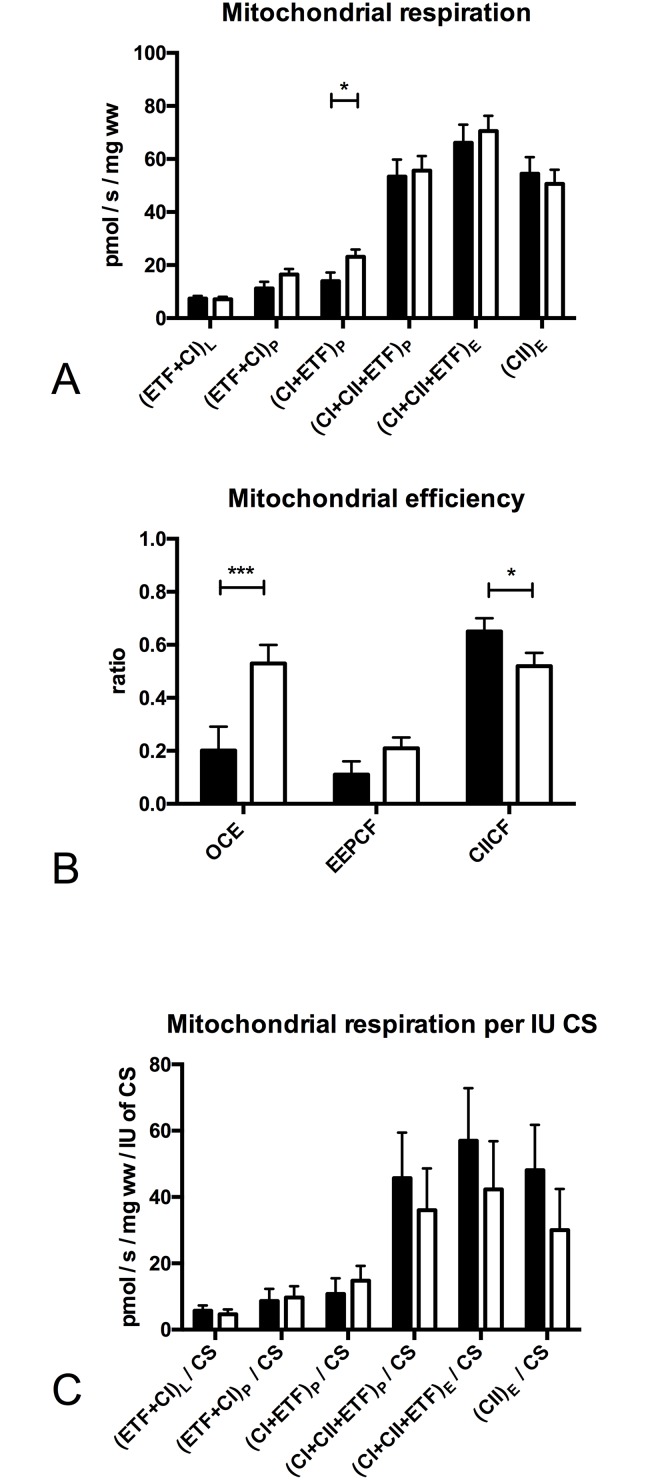
Mitochondrial respiration, efficiency and mitochondrial respiration per international unit of citrate synthase activity at baseline. Mitochondrial respiration (pmol O_2_/s/ mg wet weight of muscle fibers). **(A)** (ETF+CI)L is the LEAK state electron transfer through electron transferring flavoprotein (ETF) and complex I after addition of the substrates octanoylcarnitin (0.2mM) + malate (2mM), in the absence of ADP; (ETF+CI)P is fatty acid oxphos capacity after addition of ADP (2.5mM); (CI+ETF)_P_ is electron transfer through complex I and ETF reaching complex I oxphos capacity after addition of glutamate (10mM); (CI+II+ETF)P is electron transfer through complex I, II and ETF reaching complex I and II oxphos capacity after addition of succinate (10mM) and ADP (5mM); (CI+II+ETF)E is electron transfer through complex I, II and ETF reaching ETS capacity after FCCP titrations (0.5M max. 3M) to uncouple oxidation from phosphorylation; (CII)_E_ is ETS capacity supported by complex II after addition of rotenone (0.5M), which inhibits complex I. The subscripts L,P,E indicate the LEAK state, OXPHOS and ETS capacity **(B)** Mitochondrial efficiency at baseline: OCE: oxphos coupling efficiency (1 - (ETF+CI)_L_ / (ETF+CI)_P_); EEPCF: Excess ETS-phophorylation capacity factor (1 - (CI+CII+ETF)_P_ / (CI+CII+ETF)_E_), CII CF: complex II control factor (1 - (CI+ETF)_P_ / (CI+CII+ETF)_P_) **(C)** Mitochondrial respiration per international unit of citrate synthase (CS). Black (patients with PVD n = 11), White (healthy older adults n = 11). Values are mean and standard error of the mean, comparison between groups, Significant differences between groups * Significantly different between groups (P < 0.05), ** Significantly different between groups (P < 0.01), *** Significantly different between groups (P < 0.001)

Mitochondrial respiration supported by (CI+ETF)_P_ was 40% lower (p = 0.031, Fig 2A) in patients with PVD. Oxphos coupling efficiency was 2.5 fold lower (p = 0.004, Fig 2B) while the complex II control factor was 10% higher (p = 0.041, Fig 2B) in patients with PVD. Citrate synthase (CS) activity and mitochondrial respiration corrected for CS activity (Fig 2C) were not significantly different between groups at baseline.

### Mitochondrial response after exercise in patients with Peripheral Vascular Disease

**Fig 3:**
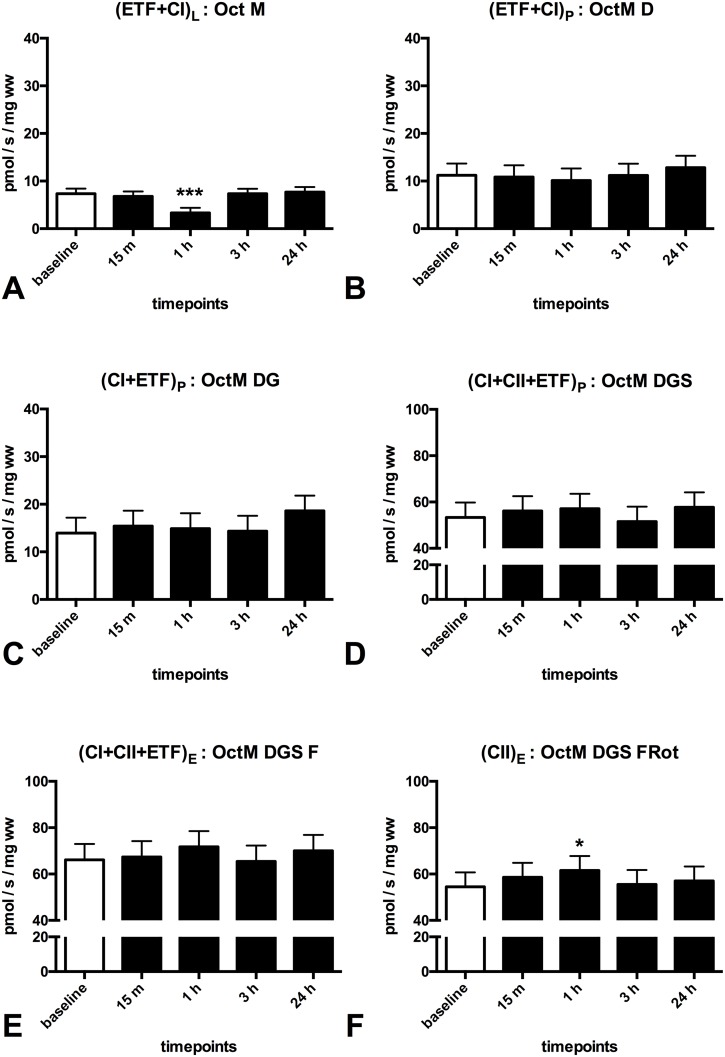
Effect of one session of calf raises upon mitochondrial respiration in patients with PVD. Mitochondrial respiration (pmol O_2_/s/ mg wet weight of muscle fibers) in patients with PVD compared to at baseline. **(A)** (ETF+CI)L is the LEAK state electron transfer through electron transferring flavoprotein (ETF) and complex I after addition of the substrates octanoylcarnitin (0.2mM) + malate (2mM), in the absence of ADP **(B)**: (ETF+CI)P is fatty acid oxphos capacity after addition of ADP (2.5mM) **(C)**: (CI+ETF)_P_ is electron transfer through complex I and ETF reaching complex I oxphos capacity after addition of glutamate (10mM) **(D)**: (CI+II+ETF)P is electron transfer through complex I, II and ETF reaching complex I and II oxphos capacity after addition of succinate (10mM) and ADP (5mM) **(E)**: (CI+II+ETF)E is electron transfer through complex I, II and ETF reaching ETS capacity after FCCP titrations (0.5M max. 3M) to uncouple oxidation from phosphorylation **(F)**: (CII)_E_ is ETS capacity supported by complex II after addition of rotenone (0.5M), which inhibits complex I. The subscripts L,P,E indicate the LEAK state, OXPHOS and ETS capacity. Oct (0.2mM octanoylcarnitin), M (2mM malate), D (2.5mM ADP),G (10mM glutamate), S (10 mM succinate), D (5mM ADP), F (0.5M FCCP titrations) Rot (0.5 M Rotenon). White (baseline); black (pst-exercise). Values are mean and standard error of the mean, n = 11. * Significantly different from pre (P < 0.05), ** Significantly different from pre (P < 0.01), *** Significantly different from pre (P < 0.001).

**Fig 4:**
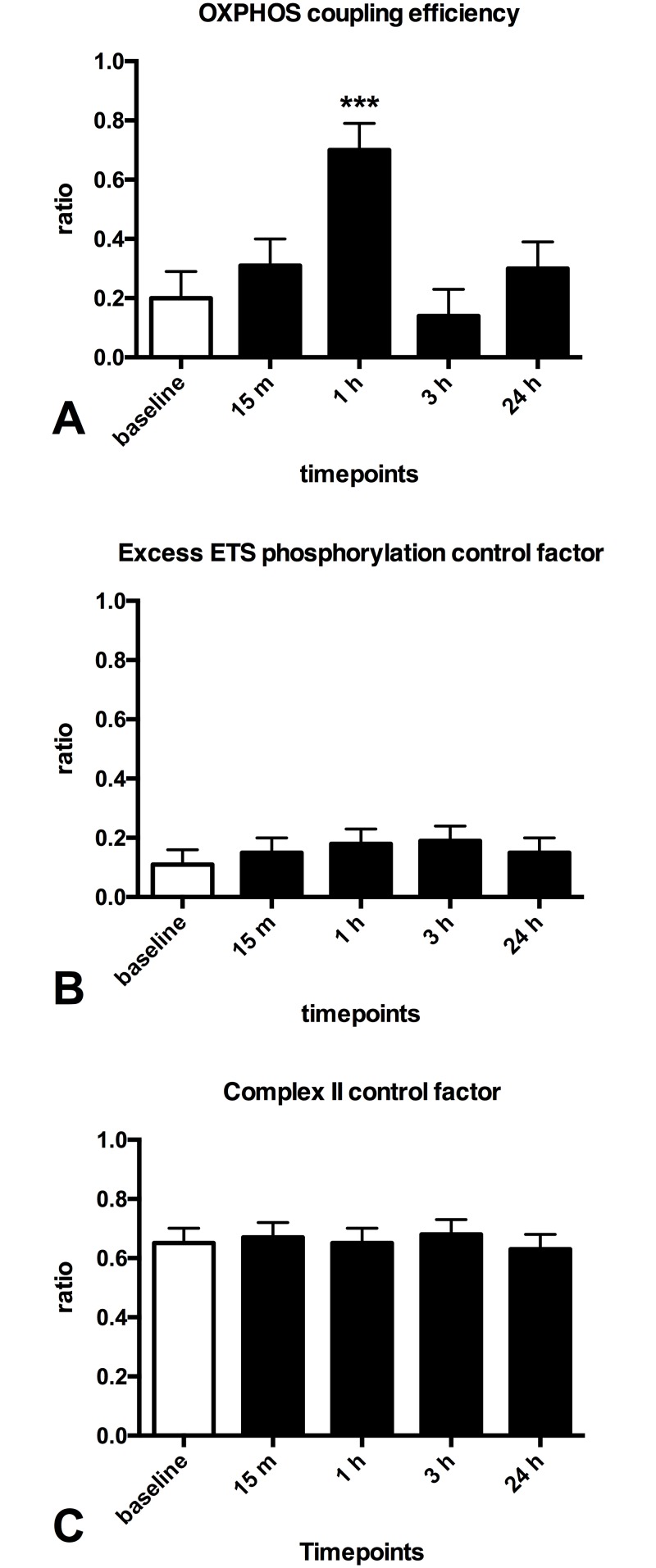
Effect of one session of calf raises upon mitochondrial function in patients with PVD. **(A)**: oxphos coupling efficiency (1 - (ETF+CI)_L_ / (ETF+CI)_P_) **(B)**: Excess ETS-phophorylation capacity factor (1 - (CI+CII+ETF)P / (CI+CII+ETF)_E_) **(C)**: complex II control factor (1 - (CI+ETF)_P_ / (CI+CII+ETF)P)White (baseline); black (post-exercise). Values are mean and standard error of the mean, n = 11. * Significantly different from pre (P < 0.05), ** Significantly different from pre (P < 0.01), *** Significantly different from pre (P < 0.001).

After performing a median of 65 calf raises patients with PVD showed a 40% decrease in mitochondrial respiration supported by (ETF+CI)_L_ in the LEAK state (p = 0.000, Fig 3A) and an increase in mitochondrial respiration supported by complex II (CII)_E_ in the ETS state at 1 hour post-exercise (~12%, p = 0.021, Fig 3F). Furthermore a 3.5 fold increase in oxphos coupling efficiency (p = 0.000, Fig 4A) was found at 1 hour post-exercise.

### Mitochondrial response after exercise in healthy older adults

**Fig 5:**
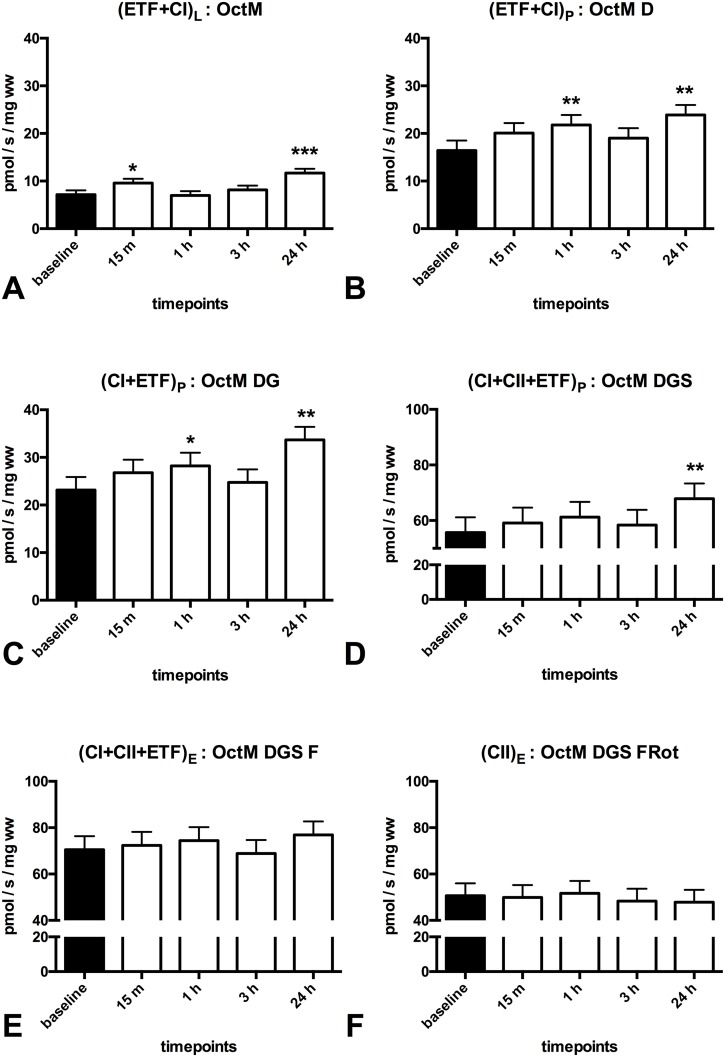
Effect of one session of calf raises upon mitochondrial respiration in healthy older adults. Mitochondrial respiration (pmol O_2_/s/ mg wet weight of muscle fibers) in patients with PVD compared to at baseline. **(A)** (ETF+CI)L is the LEAK state electron transfer through electron transferring flavoprotein (ETF) and complex I (CI) after addition of the substrates octanoylcarnitin (0.2mM) + malate (2mM), in the absence of ADP **(B)**: (ETF+CI)P is fatty acid oxphos capacity after addition of ADP (2.5mM) **(C)**: (CI+ETF)_P_ is electron transfer through complex I and ETF reaching complex I oxphos capacity after addition of glutamate (10mM) **(D)**: (CI+II+ETF)P is electron transfer through complex I, II and ETF reaching complex I and II oxphos capacity after addition of succinate (10mM) and ADP (5mM) **(E)**: (CI+II+ETF)E is electron transfer through complex I, II and ETF reaching ETS capacity after FCCP titrations (0.5M max. 3M) to uncouple oxidation from phosphorylation **(F)**: (CII)_E_ is ETS capacity supported by complex II after addition of rotenone (0.5M), which inhibits complex I. The subscripts L,P,E indicate the LEAK state, OXPHOS and ETS capacity. Oct (0.2mM octanoylcarnitin), M (2mM malate), D (2.5mM ADP),G (10mM glutamate), S (10 mM succinate), D (5mM ADP), F (0.5M FCCP titrations) Rot (0.5 M Rotenon). Black (baseline); white (post-exercise). Values are mean and standard error of the mean, n = 11. * Significantly different from Baseline (P < 0.05), ** Significantly different from Baseline (P < 0.01), *** Significantly different from Baseline (P < 0.001)

**Fig 6:**
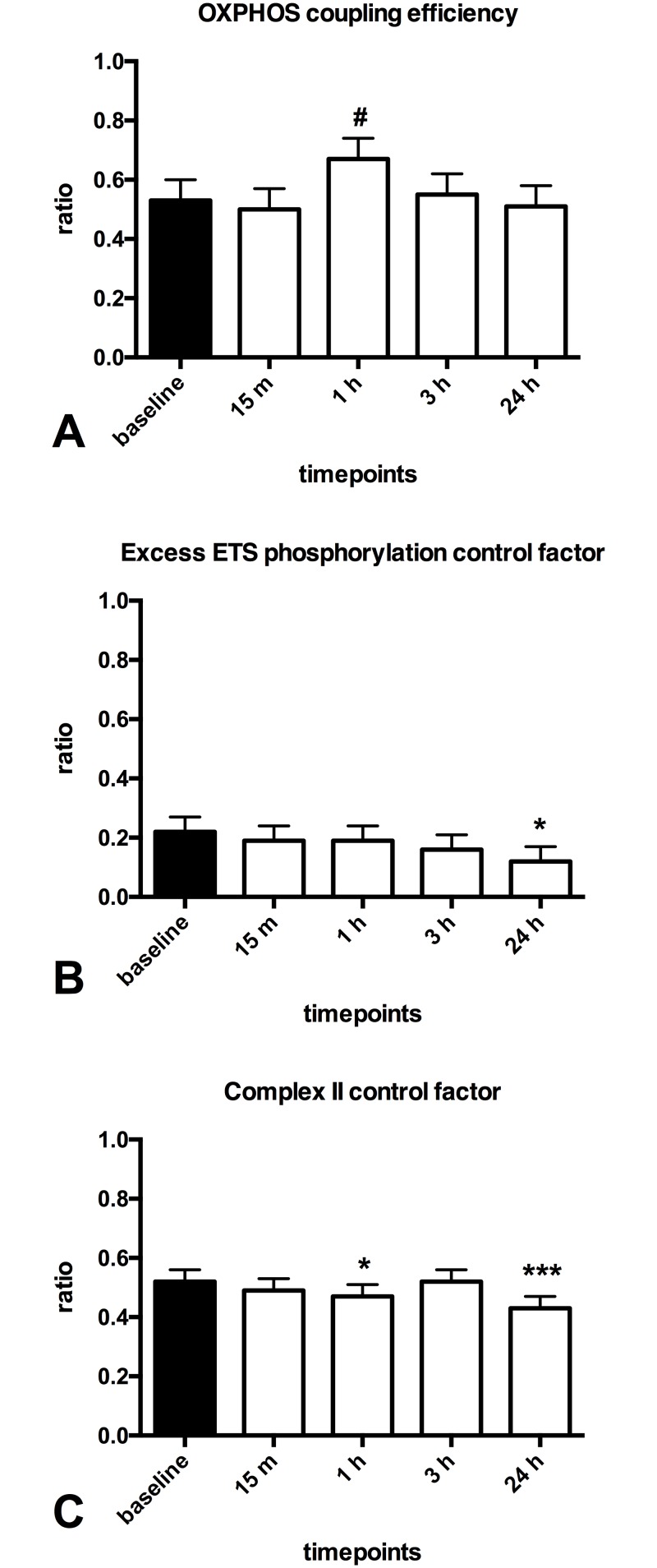
Effect of one session of calf raises upon mitochondrial function in healthy older adults. **(A)**: oxphos coupling efficiency (1 - (ETF+CI)_L_ / (ETF+CI)_P_) **(B)**: Excess ETS-phophorylation capacity factor (1 - (CI+CII+ETF)P / (CI+CII+ETF)_E_) **(C)**: complex II control factor (1 - (CI+ETF)_P_ / (CI+CII+ETF)P)Black (baseline); white (post-exercise). Values are mean and standard error of the mean, n = 11. # trend to a significant difference (P<0.1),* Significantly different from pre (P < 0.05), ** Significantly different from pre (P < 0.01), *** Significantly different from pre (P < 0.001)

The healthy older adults performed 100 calf raises without any pain. They increased mitochondrial respiration supported by (ETF+CI)_L_ at 15 minutes (~35%, p = 0.020, Fig 5A) post-exercise. At one hour post exercise increased respiration supported by (ETF+CI)_P_ (~25%, p = 0.002, Fig 5B) and (CI+ETF)_P_ (~20%, p = 0.025, Fig 5C) were shown. While at 24 hours post-exercise increased respiration supported by (ETF+CI)_L_ (~60%, p = 0.000, Fig 5A), (ETF+CI)_P_ (~45%, p = 0.000, Fig 5B), (CI+ETF)_P_ (~40%, p = 0.000, Fig 5C) and (CI+CII+ETF)_P_ (~23%, p = 0.000, Fig 5D) were shown. A decreased complex II control factor was found (~20%, p = 0.037, Fig 6C) at one hour and at 24 hours (~25%, p = 0.000, Fig 6C) post-exercise. At 24 hours also a decreased excess electron transfer system phosphorylation capacity factor (~45%, p = 0.048, Fig 6B) was found.

### Sensitivity analyses and mixed linear models

None of the sensitivity analyses influence within group comparisons post-exercise. Through maximal value and last observation carried forward sensitivity analyses the significant difference between groups in respiration supported by complex I at baseline is lost.

## Discussion

The current study confirms the concept of defective mitochondrial function as a pathophysiologic component of peripheral vascular disease by demonstrating an impaired mitochondrial respiration supported by complex I (with further electron transfer through complex III and IV) at baseline. This is in line with previous studies that show impaired mitochondrial enzyme activity and mitochondrial respiration of complex I, complex III and complex IV in calf muscle mitochondria of patients with PVD and also in mice with ligated femoral arteries [3–5,17]. Notably, the only electron transfer system complex that seems not to be reduced in skeletal muscle of patients with PVD is complex II [3,4]. Our finding that complex II control factor was higher in patients with PVD at baseline supports this view. Complex II control factor represents a functional aspect of complex II and indicates the fractional change of respiration after addition of the complex II substrate succinate. Interestingly, mitochondrial adaptations after a single session of calf raise exercise in patients with PVD were restricted to respiration supported by complex II at 1 hour post-exercise. Complex II is the only protein complex in the electron transfer system that is encoded by nuclear DNA and not mitochondrial DNA [18,19]. Since other complexes are impaired in patients with PVD this supports the notion that mitochondrial DNA might be compromised in peripheral vascular disease [20–22]. Several studies imply ROS production as a link between mitochondrial DNA damage and mitochondrial respiration [23,24]. Even though ROS production was not measured in the present study, an increased oxphos coupling efficiency in patients with PVD one hour post-exercise suggests a tighter coupled electron transfer system, possibly resulting in elevated ROS. Hence, the observed mitochondrial adaptation in response to one session of exercise in patients with PVD might be the result of nuclear- rather than mitochondrial adaptation.

Exercise induces ischemia while having pain and reperfusion starts at rest in patients with PVD [25]. It has been imposed that repetitive cycles of exercise until pain followed by rest may be important for adaptation to take place in patients with PVD to increase functional capacity [26–28]. The patients tested in the current study performed calf raises until pain was felt in the calf musculature; this was followed by five extra repetitions and than rest. Hence, it can be assumed that ischemia and reperfusion of the gastrocnemius muscle took place. In patients with PVD we showed only changes related to respiration supported by complex II after one session of exercise. Complex II plays a crucial role in mitochondrial adaptation to ischemia followed by reperfusion of the gastrocnemius muscle of rats [10]. Ischemia in rat hearts increases succinate directly proportional to the time of ischemia. During reperfusion increase complex II activity (succinate dehydrogenase) reverses the increased succinate [29]. There are major differences compared to the methods applied in our exercise study and the studies upon ischemia and reperfusion (preconditioning). However, our data also demonstrated that respiration supported by complex II is increased. Repetitive cycles of pain induced by physical training increase activity of complex IV in both patients with PVD and rats with ligated femoral arteries [3,30,31]. Repeating the calf raise exercise might provoke chronic adaptations in the calf muscle leading to increased mitochondrial respiration supported by complex II.

We found an increase in mitochondrial respiration supported by electron transfer flavoprotein and complex I at one hour and at 24 hours post-exercise in the healthy older adults. This indicates increased fatty acid oxidation of octanoylcarnitin. Previous studies demonstrate that fatty acid oxidation increases after one session of exercise. One study demonstrates decreased plasma fatty acid concentrations at one hour combined with increased muscle lipase activity at 24 hours after one session of running in healthy young adults [32]. In untrained healthy obese adults fatty acid oxidation increases up to one hour following 60 minutes of moderate intensity exercise on a cycle ergometer [33]. Both these studies indicate a shift towards lipid metabolism in the first hour post-exercise in healthy individuals. Furthermore, the mitochondrial response to one session of calf raise exercise in healthy older adults seems to be in line with a previous study demonstrating increased mRNA expression of cytochrome c oxidase subunit-IV (COX IV; part of complex IV) and cytochrome c in the gastrocnemius muscle of mice after one session of treadmill exercise running for 90 minutes [34]. Expression of mRNA increases first at 1 hour and later at 6 and 12 hours post-exercise. In older adults one session of 20-minute high intensity interval training increases mRNA of COX IV expression at 3 hours post-exercise, further followed by increase in COX IV proteins 3 days later [7]. Other studies demonstrate an increase in COX IV protein already at 24 hours post-exercise [9,35]. Repetitive sessions of high intensity exercise over a 2-week period further stimulate fatty acid oxidation and mitochondria, by increasing the activity of the enzyme 3-hydroxyacyl-CoA dehydrogenase and abundance of COX IV proteins [35]. By repeating calf raise exercise chronic adaptations in the calf muscle of healthy older adults might take place leading to increased mitochondrial respiration.

### Limitations

In this study we have mainly concentrated our evaluation upon the different response within each group separately. Comparisons between groups at baseline should therefore be interpreted with caution. Also the workload of the calf raise exercise is difficult to compare with other exercise studies. The mechanisms for the mitochondrial responses to exercise in PVD still need to be resolved.

## Conclusion

We demonstrate that respiration supported by complex I was reduced in patients with PVD and that complex II control factor was higher at baseline. Based on our findings a shift towards respiration supported by complex II is suspected to be a pathophysiologic component of peripheral vascular disease. Furthermore, one session of calf raises increased mitochondrial respiration only supported by complex II at one hour post-exercise in patients with PVD. In contrast, healthy older adults showed increased mitochondrial respiration supported by electron transfer flavoprotein and complex I at one hour and 24 hours post-exercise.

In summary, we show that both patients with PVD and healthy older adults increase mitochondrial respiration already after one session of calf raises. To our best knowledge this is the first study describing the time course of mitochondrial respiration in response to one session of exercise in patients with PVD.

## Supporting Information

S1 Checklist TREND statement checklist. (PDF)

S1 Protocol Protocol as approved by Ethical committee. (PDF)
