# Supplementary material for: Mitochondrial Respiration after One Session of Calf Raise Exercise in Patients with Peripheral Vascular Disease and Healthy Older Adults
Source: PLoS One. 2016 Oct 19;11(10):e0165038. doi: 10.1371/journal.pone.0165038 (PMC5070763; doi:10.1371/journal.pone.0165038)
Supplement: S1 Protocol — (PDF) [file pone.0165038.s002.pdf]

‘The initial effect of 5$^+$ training on mitochondrial function in patients with intermittent claudication`

Ph D candidate Michel van Schaardenburgh

Project ‘Five plus’

Circulation Research Group
ISB, Faculty of Medicine
Norwegian University of Science and Technology (NTNU)
N-7491 Trondheim, Norway
Phone: +47 (728)29759

Contents

Aim ... 3
Background and theoretical foundations ... 3
Energy metabolism in the muscle ... 4
Mitochondrial respirometry ... 5
Study plan ... 6
Study design ... 7
Inclusion/exclusion criteria patients with intermittent claudication ... 7
Inclusion/exclusion criteria control group ... 8
Polyclinic ... 9
'Generation 100' control group ... 9
Testing capacity ... 10
Testing dates ... 10
Research unit ... 11
Timeschedule ... 11
Gastrocnemius muscle biopsy: ... 12
Laboratory: ... 13
High-resolution respirometry ... 14
Citrate synthase activity measurement ... 15
Statistical analysis and sample size ... 15
Scientific impact ... 16
Ethical perspectives ... 16
Literature ... 17

Aim

This study describes a clinical trial designed to test the hypothesis that one bout of a "Five plus training"(5^+) training induces changes in qualitative and quantitative response in the mitochondria. This will be based on measurements at different time intervals throughout the day following the training intervention.

We define the qualitative response as being the efficiency of extracting and using oxygen by the different complexes of the electron transfer system (oxidative phosphorylation). This will be explained later. We characterize the quantitative response as the maximal oxygen extraction through the total process of oxidative phosphorylation in the mitochondria.

The results will give us an insight about the frequency and timing of 5^+ training required to induce the optimal mitochondrial response.

Background and theoretical foundations

In this study we will concentrate on the initial effect on patients with intermittent claudication from one bout of resistance training that we have entitled 5^+. 5^+ training consists of standing in front of a wall which is used for hand support and balance. The body will be lifted with the calf musculature to the maximal height that the subject can achieve. This will be repeated until pain is felt in the musculature. Following the initiation of pain the subject will perform five extra repetitions. Since this training only utilizes the body weight, the training can be performed at home without additional equipment or cost.

In this way, there is a natural progression of training dose as muscular adaptation occurs. The five extra raises secure induction of ischemia and reperfusion. This is equal to preconditioning.

In patients with peripheral arterial disease, increasing the workload causes an inequality in the supply of and demand for oxygen in the mitochondria. Aerobic generation of adenosine tri phosphate (ATP) in the

mitochondria becomes insufficient and anaerobic metabolism predominates. This results in increased lactic acid production. During this anaerobic process also hydrogen (H^+) in the cells increases leading to a decreasing pH-level in the cell. As a consequence symptoms of intermittent claudication evolve.[1]

Mitochondria in patients with peripheral arterial disease (PAD) do not have the capacity to produce as much ATP as normal muscle mitochondria. [1, 2] Patients with PAD find themselves in double jeopardy; not only do they have a decreased supply of nutrients and oxygen, as a result of diseased arteries, but the concurrent mitochondrial respiratory defect leads to an even lower ATP production from the amount of O_2 present in spite of the atherosclerotic disease. [3, 4]

Energy metabolism in the muscle

Skeletal muscles are dependent on adenosine tri phosphate (ATP). ATP is the molecule that is used for intracellular energy transfer. ATP is an end product of cellular respiration and is used by enzymes and structural proteins in many different cellular processes.

ATP is mostly produced in the inner part (matrix) of the mitochondria through the citric acid cycle and oxidative phosphorylation (OXPHOS). Mitochondria are therefore also called 'the power plant' of the cell. Energy is build up over the inner membrane through electrons that pass a series of complexes (I, II, III and IV), collectively also known as the electron transfer system. (See figure 1)

At complex I nicotinamide adenine dinucleotide hydroxide (NADH), a product from the citric acid cycle, is reduced and divided into NAD^+ and a hydrogen (H^+). During this process electrons are passed from NADH over to complex I and a H^+ molecule is transported over from the matrix to the intermembrane space. Also at complex III and IV H^+ is moved to the intermembrane space. At the end of the electron transports chain H^+ transfers back into the matrix through ATP synthase. The energy that is build up over the inner membrane is transferred over to ATP.[5] This process is called oxidative phosphorylation (OXPHOS) and it is dependent on oxygen for its functioning. Oxygen is used at complex IV as seen in figure 1.

Mitochondrial respirometry

Through mitochondrial respirometry you are able to measure the efficiency of oxidative phosphorylation. [6, 7] Mitochondria are isolated from their surroundings making it possible to investigate the biochemical processes within this subcellular component.

By adding different substrates, as described later, to an oxygraph-2k chamber the different complexes can be investigated by blocking or inducing redox reactions. By doing this we get an insight in the qualitative aspects of mitochondrial respiration. [8, 9]

Respirometry measures quantitative and qualitative aspects of oxidative phosphorylation. The maximal respiration rate, a quantitative measurement, is assessed. This appraises to which extent oxygen is used, in relation to the energy that is transferred into ATP.

To control mitochondrial content we test a commonly used quantitative enzyme marker, Citrate synthase. Citrate synthase is a pace-making enzyme in the first steps of the citric acid cycle (Krebs cycle) and is localized in the mitochondrial matrix.

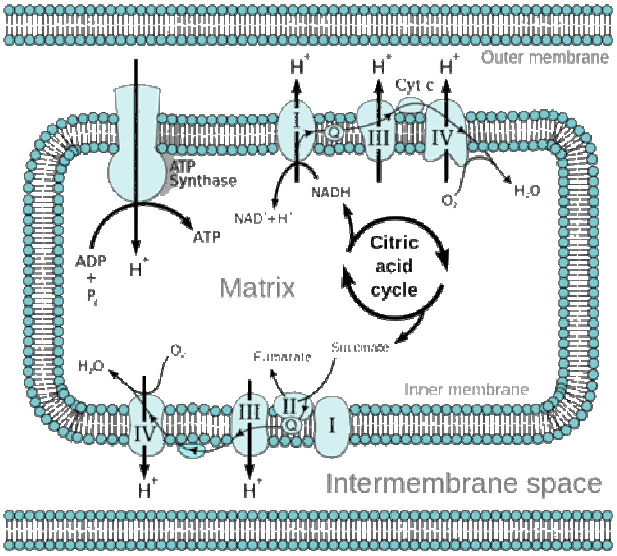

Figure 1: Oxidative phosphorylation (OXPHOS) and electron transfer system in the mitochondria.

Current evidence does not support the hypothesis that training leads to a significant improvement in blood flow.[1] Explanations for improvements are linked to metabolic changes and increased oxidative capacity of skeletal muscles.[3, 10, 11]

Resistance training increases the qualitative and quantitative aspects of mitochondria by increasing the efficiency of the oxidative energy transfer.[12, 13]

Ischemic preconditioning (IPC) seems to stimulate cellular metabolism during recovery from ischemia by a higher efficiency of oxidative phosphorylation. [14]

It has been shown that High Intensity Training (HIT) increases mitochondrial protein content and enzyme activity in the muscle vastus lateralis tissue 24 hours after the HIT. [15]

The question arises whether improved mitochondrial function can partly compensate for less O_2 and improve the clinical situation in patients with intermittent claudication (IC).

Study plan

In this study we will concentrate on the initial reaction of the muscle of interest (gastrocnemius) on one bout of 'five plus'.

We hypothesize that one bout of 5^+ *gives qualitative changes through a more effective energy transfer, over to the membrane, during oxidative phosphorylation and quantitative changes shown by a higher total oxygen use.*

Study design

PAD is divided into 3 levels of arterial insufficiency.

1. Asymptomatic PAD; ABI under 0.9: patients do not experience any specific symptoms.
2. Intermittent claudication (IC); IC is characterized by repeated muscle discomfort in the lower limb reproduced by exercise and relieved by rest within 10 minutes. Patients may complain of muscle fatigue, aching or cramping. Ankle-brachial index is between 0.4 and 0.9. [10]
3. Critical limb ischemia is described as a decrease in limb perfusion that causes a potential threat to its viability. The condition includes non-healing ulcerations, ischemic rest pain or pedal gangrene. Ankle-brachial index is usually between 0 and 0.4. [10]

Patients will be recruited by the vascular surgeons at the vascular unit of the surgical department, St Olavs Hospital in Trondheim.

The control group will be recruited from the ´Generation 100` study. During the randomization in this study a third of the participants will be randomized into the control group.
Participants that are randomized to the control group will so be informed about the Five Plus study and asked if they are willing to participate. When given consent they can be included in the control group of our study Five Plus.

Inclusion/exclusion criteria patients with intermittent claudication

Patients will be included if they meet the following criteria.

- A history of any type of uni- or bilateral exertion leg pain.
- A maximal claudication distance that is less than 300 meter
- Diagnosed with intermittent claudication secondary to vascular insufficiency
- An ankle-brachial index between 0.4 and 0.9.
- If a vascular intervention is planned the patient can still be included
- Has to be able not to smoke during the testing day
- Lives in Trondheim

Patients will be excluded in the case of the following conditions;

- Diagnosed with critical limb ischemia
- ABI >0.90 or < 0.4
- Limited exercise tolerance
- Heavy smoker
- Diabetes mellitus
- Underwent a vascular intervention in the last 3 months
- Active cancer, renal- or liver disease.

Inclusion/exclusion criteria control group

Participants from generation 100 will be included if they meet the following criteria.

- Has to be able not to smoke during the testing day
- Lives in Trondheim

Patients will be excluded in the case of the following conditions;

- A history of any type of uni- or bilateral exertion leg pain.
- ABI <0.90
- Heavy smoker
- Active cancer, renal- or liver disease.
- Diabetes mellitus

Polyclinic

When a potential patient is presented for the study the Ph. D. candidate will be called upon.

He can be reached on one of the following numbers 72829759 or 97431394.

If the candidate is available he will inform the patient about the project. This conversation will take place at the polyclinic. The patient will be seated in the waiting room until a proper room is found.

Clinical information will be taken from electron patient journal (Doculive).

A clinical research form (CRF) will be filled out by the researcher. On the CRF the following variables will be notified; social security number, telephone number, age, sex, smoking, hypertension, dyslipidemia, coronary artery disease, obesity, medication, ankle brachial index, medical imaging done, grade and level of obstruction (above inguinal ligament, above- or below knee).
This CRF form is to be found in the back of a blue binder.

When informed, the patient has to give written consent. After this the patient can be included into the study. It is stated that the patient can stop the participation in the study whenever he or she wants.

If the researcher is not able to answer the telephone the patient will later be contacted by phone on Monday or Friday morning. Therefore the following information about the patient will be written down by a nurse at the polyclinic and collected in the back of the blue binder.

1. name
2. social security number
3. telephone number

When leaving the polyclinic the patient will be given a patient information document and shown where to sign when giving consent. When the patient has not written consent, when leaving the polyclinic, he/she will be invited to a meeting with the researcher.

‘Generation 100’ control group

During week 14 and 17 participants randomized to the control group of generation 100 will be presented to the Ph. D. candidate directly after randomization. A patient information form is given to the participant of the control group which he or she can read while waiting for the candidate. He will so inform the participant personally.

Letters will be send to participants already randomized to the control group. These will be contacted by telephone and invited for an interview.

During the interview a clinical research form (CRF) will be filled out by the researcher. On the CRF the following variables will be notified; social security number, telephone number, age, sex, smoking, dyslipidemia, hypertension, dyslipidemia, coronary artery disease, obesity, medication and ankle brachial index.

This CRF form is to be found in the back of a red binder.

Both the red and the blue binder will be in close proximity of the researcher. When stored they will be in a closed locker which only can be opened by the researcher.

Testing capacity

This research project will have a testing capacity to include 32 patients. The capacity of one week of testing varies from 2 to 4 patients. Every patient is tested over two days. And two patients can be tested at the same time.

Testing dates

**Week 17**	22.04.13	Monday
	23.04.13	Tuesday
	24.04.13	Wednesday
	25.04.13	Thursday
**Week 20**	14.05.13	Tuesday
	15.05.13	Wednesday
**Week 21**	20.05.13	Monday
	21.05.13	Tuesday
	22.05.13	Wednesday
	23.05.13	Thursday
**Week 22**	27.05.13	Monday
	28.05.13	Tuesday
	29.05.13	Wednesday
	30.05.13	Thursday
**Week 23**	03.06.13	Monday

	04.06.13	Tuesday
	06.06.13	Thursday
	07.06.13	Friday
**Week 24**	10.06.13	Monday
	11.06.13	Tuesday
	12.06.13	Wednesday
	13.06.13	Thursday
**Week 25**	17.06.13	Monday
	18.06.13	Tuesday
	19.06.13	Wednesday
	20.06.13	Thursday
**Week 26**	25.06.13	Monday
	26.06.13	Tuesday
	27.06.12	Wednesday
	28.06.12	Thursday

When the patient has given consent he will be sent a letter within 2-3 weeks in which the following information will be stated;

1. On which date the testing will take place
2. Where he will have to meet (research unit, first floor Acute Heart Lung building, St Olavs)
3. That it is not allowed to smoke during the testing day
4. The time schedule of the day
5. That there will be served a standard lunch
6. That the patient will attend fasting (also no coffee)
7. That a doctor will be present

Research unit

The first subject is expected to come to the research unit at 08:15. The subject has to be in a fasting state two hours prior to arrival. The researcher and nurse will give a new introduction. The patient will be informed about what to expect on this testing day. A first biopsy is taken 08.45 followed by one bout of $5^{+.}$ After this 4 biopsies will be taken; one at 15 minute, 1 hour, 3 hour, and one at 24 hours on the second day.

The second subject is expected at the research unit at 09:30 and will follow the same program as stated above.

After the five plus bout the patients are instructed to be as least active as possible. He or she is not allowed to go up a staircase during the testing day. The participants are not allowed to smoke during the testing period.

The patients will be served a standard lunch and coffee (without caffeine) during the day.

Timeschedule

Tid	DAY 1		Tid	DAY 2	
08:15:00	Introduction Subject 1		08:15:00		
08:30:00			08:30:00	Subject 1	
08:45:00	biopsy 1		08:45:00		
09:00:00	**Five pluss**		09:00:00	biopsy 5	
09:15:00	biopsy 2		09:15:00		
09:30:00		Introduction Subject 2	09:30:00		
09:45:00			09:45:00		
10:00:00	biopsy 3		10:00:00		Subject 2
10:15:00		biopsy 1	10:15:00		
10:30:00		**Five pluss**	10:30:00		biopsy 5
10:45:00		biopsy 2			
11:00:00	Lunsj				
11:15:00					
11:30:00		biopsy 3			
11:45:00					
12:00:00	biopsy 4				
12:15:00					
12:30:00	Avslutning	Lunsj			
12:45:00					
13:00:00					
13:15:00					
13:30:00		biopsy 4			
13:45:00					
14:00:00		Avslutning			

Gastrocnemius muscle biopsy:

We will use a minimal invasive procedure; a microbiopsy technique, as described by Votion [9] and Hayot[16]. Briefly the sampling site will be shaved; a stamp with six holes (figure 2) will be pressed on the skin over the lateral part of the gastrocnemius muscle so that the biopsy placement will be standardized. (Only five biospies will be taken). These points will be marked with a permanent marker (Edding Control 79, Germany). The skin will be sterilized with chlorhexidine 5% and locally anesthetised by subcutaneous injection of Marcain with adrenalin (Astra Zeneca, Oslo, Norway). This local anesthetic will be strictly injected under the skin, so that alteration of muscle mitochondrial energetics will not be induced.

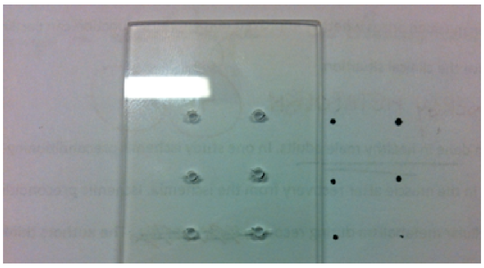

Figure 2: standardisation stamp

A 14 gauge (diameter 1.628 mm) insertion cannula (Biopince, Medical device technologies inc., Gainesville, Florida USA) will puncture the skin perpendicular to the muscle until the fascia is pierced. Muscle biopsy samples will be obtained with a sterile 16 gauge (diameter of 1,291 mm) biopsy needle. (Biopince, Medical device technologies inc., Gainesville, Florida USA)

Muscle micro biopsy specimens will be taken at 13 mm depth. In short, a muscle sample is obtained by the activation of a trigger button, which unloads the spring and activates the needle to collect a muscle piece. The biopsy needle is then slid out of the insertion cannula while the latter is maintained in place. The muscle specimen is removed from the biopsy needle using a sterile scalpel. [16]

Samples will be taken at every biopsy interval. The skin will be closed using Steri-Strips (3M, Skjetten, Norway).

The muscle tissue [6, 9, 13, 17] will be immediately transferred into ice-cold relaxing medium (BIOPS) containing 10 mmol/l Ca^{2+} /EGTA buffer.

Laboratory:

The researcher will start the O2k respirometry 2 hours prior to analysis so that it is calibrated in time.

On day 1 the analyzer will fetch the biopsies at the research unit at 10:15 and 12:30.

Biopsy 4 and biopsy 5 will be analyzed on day two and taken to the laboratory by the researcher.

All data is depersonalized (patient 1,2 etc.) and saved both on the DatLab software (Oroboros Instruments) on the desk computer and on a USB stick that serves as a backup, which is stored in a closed locker separate of the blue and red binder.

The following folders will be used:

Patient group

..://five plus/PAD/O2k analysis/subject(1,2,...10) / biopsy (1,2 etc.).

..://five plus/PAD/citrate synthase /subject(1,2,...10) / biopsy (1,2 etc.)

Control group

..://five plus/PAD/O2k analysis/subject(1,2,...10) / biopsy (1,2 etc.).

..://five plus/PAD/citrate synthase /subject(1,2,...10) / biopsy (1,2 etc.)

The laboratory will follow these procedures:

A small sample of muscle tissue will be transferred into BIOPS onto a small petri dish on an ice-cold metal plate and the muscle fibers will be separated using forceps with sharp tips. To ensure complete permeabilization the fibers will be incubated by gentle agitation at 4°C in BIOPS solution containing 50µ g/ml saponin for 20 min. Fibers will be washed for 10 min at 4°C in mitochondrial respiration medium [MiR05; 0.5 mM EGTA, 3 mM MgCl2 K-lactobionate, 20mMtaurine, 10mMKH2, 60mM PO4, 20mMHEPES, 110 mM sucrose and 1 g/l BSA essentially fatty acid free, adjusted to pH 7.1], and wet weight of the fibers (between 1 and 2 mg is necessary) will be measured on a microbalance Sartorius (VWS, Oslo, Norway). With this method, the cholesterol-rich plasma membrane is selectively permeabilized, leaving intracellular membrane structures, such as mitochondria intact, and the entire mitochondrial population can be studied in the muscle sample

High-resolution respirometry.

One sample will be used per respirometer chamber (Oxygraph-2k; Oroboros Instruments, Innsbruck, Austria) containing 2,5 ml MiR05 at 37°C. Oxygen concentration ($\mu M = \frac{nmol}{ml}$) and oxygen flux (pmol·s^{-1}·mg^{-1}; negative time derivative of oxygen concentration, divided by muscle mass per volume) will be recorded using DatLab software (Oroboros Instruments). The Oxygraph-2k is a two-chamber titration-injection respirometer with a limit of detection of volume-specific oxygen flux of 1pmol·s^{-1}·ml^{-1}. Three instruments will be operated in parallel. Instrumental background oxygen flux will be corrected online, accounting for sensor oxygen consumption and oxygen diffusion between the medium and the chamber boundaries. The oxygen concentration in the chamber will be maintained between 250 and 500 μ M to avoid oxygen limitation of fiber respiration. Intermittent reoxygenations will be achieved by injecting 200 mM hydrogen peroxide solution into the medium containing high catalase activity. In the substrate-uncoupler-inhibitor titration protocol, the following substrates (fig. 3) will be added (final concentrations): Malate (2mM) and octanoylcarnitine (0.2 mM) to support electron entry from fatty acid-oxidation through electron-transferring flavoprotein (ETF) and Complex I (CI) to coenzyme Q (2). [6] Active respiration will be stimulated by ADP (2.5 mM; lipid oxidative phosphorylation (OXPHOS) capacity or state 3). Pyruvate (5 mM) will be added to support electron entry form glycolysis-oxidation. Further addition of CI substrate glutamate (10 mM) yields reduced nicotinamide adenine dinucleotide (NADH), which feeds electrons into CI (NADH-ubiquinone oxidoreductase). Succinate (10 mM) will be added to stimulate CI+II- linked respiration, providing convergent electron input into the Q-junction simultaneously through CI (NADH) and CII (succinate). This combination of substrates is required for reconstitution of tricarboxylic acid (TCA) cycle function and represents a substrate cocktail required to achieve physiological respiratory capacity. [7] Adding another 2.5 mM ADP after glutamate or succinate resulted in ADP saturated respiration (5 mM) to obtain CI or CI+II-linked OXPHOS capacity. Cytochrome c (10 M) will be added to test the integrity of the outer mitochondrial membrane. Electron transfer system capacity (CI+IIE) will be reached by stepwise addition of the uncoupler carbonylcyanide p-trifluoromethoxyphenyl-hydrazone (FCCP) at 0.025μ M steps. The addition of rotenone (0.5 μ M; inhibitor of CI) induces a slow decline of respiration to succinate-supported ETS capacity. Sequential addition of malonic acid and antimycin A inhibits flux. [6, 13]

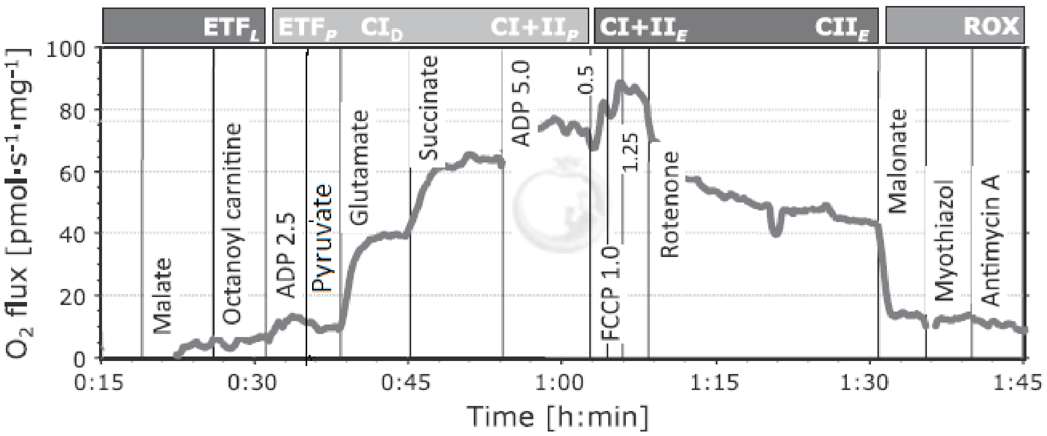

Figure 3 Substrate-uncoupler-inhibitor titration protocol (SUITS) in Five Plus study. [13]

Citrate synthase activity measurement

Citrate synthase (CS) activities will be assayed in homogenates of the skeletal muscle samples used in respiration measurements: The contents of the Oxygraph-2k chambers (2 ml each) will be removed after each respiration experiment and washed once with 2 ml of MiR05. One per cent Triton X-100™ and 2µl of a protease inhibitor cocktail (Sigma Aldrich Cat. 539134) will be added to the combined solutions (content and wash) and then homogenized for 30 sec with a homogenizer near maximum speed. The homogenate will then be centrifuged for 15 minutes at 4°C and the supernatant will be removed, frozen in liquid nitrogen, and stored at -80°C. CS activity will be measured fluorometrically at 412 nm and 25°C (Citrate Synthase Assay Kit, Sigma-Aldrich) according to manufacture. [17]

Measurement of Citrate synthase (CS) activities will take place at baseline and 24 hours after the bout of 5^+ training.

Statistical analysis and sample size

Based on a two sided t-test and an expected, based on previous studies [14, 15], change in mitochondrial activity of 20 percent, a significance level of 0.05 and a power of the test of 80%. A total of 8 patients have to be included.

We will include a total of 10 patients to get a power of 89%.
Also in the control group a total of 10 participants will be included.

Mitochondrial enzyme activity data will be analyzed using a one way ANOVA for repeated measures. Significance will be set at p value < 0.05. Data will be presented as mean SD.

Group statistics as the variables age, ankle brachial index, will be presented as mean SD. Sex, smoking, medication, medical imaging done, grade and level of obstruction will be presented in percentage.

Statistical analyses will be performed using Statistical Package for the Social Sciences Version 20 or higher edition (SPSS Inc., Chicago, IL, USA).

Scientific impact

Achieving the study objectives will have the following scientific impact:

A positive result through this research may help us to establish an original and novel therapeutic tool for the prevention and treatment of peripheral arterial disease. This might lead to reducing morbidity and social isolation.

Ethical perspectives

This clinical study will be accomplished according to the Declaration of Helsinki and be approved by the Regional Committee for Medical Research Ethics. Written and informed consent will be obtained from all subjects at the beginning of the studies.

Reduced functionality as a consequence of inactivity is an important trigger of reduced quality of life, invalidity and premature death.

Conservative treatments have shown to have good effect but supervised programs have not been established on a national basis.

Increased knowledge on how to reduce costs and resources associated with treatment would be beneficial for the whole population and is anticipated to have a vast public health and socioeconomic impact.

This project aims to identify an effective exercise programs for large patients groups in order to become an effective tool in prevention, treatment, and rehabilitation medicine, and as such provide detailed exercise training recommendations.

There are no principal ethical concerns related to research on physical exercise and conditioning.

Disadvantages are minor for the participant, but the patients may expect to be sore in the muscle where the biopsies are collected. The infection risk may also be elevated.

Literature

1. Tan, K.H., L. De Cossart, and P.R. Edwards, *Exercise training and peripheral vascular disease.* The British journal of surgery, 2000. **87**(5): p. 553-62.
2. Pernow, B., et al., *Leg blood flow and muscle metabolism in occlusive arterial disease of the leg before and after reconstructive surgery.* Clin Sci Mol Med, 1975. **49**(3): p. 265-75.
3. Pipinos, II, et al., *The myopathy of peripheral arterial occlusive disease: Part 2. Oxidative stress, neuropathy, and shift in muscle fiber type.* Vascular and endovascular surgery, 2008. **42**(2): p. 101-12.
4. Pipinos, II, et al., *Abnormal mitochondrial respiration in skeletal muscle in patients with peripheral arterial disease.* J Vasc Surg, 2003. **38**(4): p. 827-32.
5. Mathews, C.K., K.G. Ahern, and K.E. Van Holde, *Biochemistry* 2000: San Francisco, Calif. : Benjamin/Cummings.
6. Pesta, D. and E. Gnaiger, *High-resolution respirometry: OXPHOS protocols for human cells and permeabilized fibers from small biopsies of human muscle.* Methods Mol Biol, 2012. **810**: p. 25-58.
7. Gnaiger, E., *Capacity of oxidative phosphorylation in human skeletal muscle: new perspectives of mitochondrial physiology.* Int J Biochem Cell Biol, 2009. **41**(10): p. 1837-45.
8. Kuznetsov, A.V., et al., *Analysis of mitochondrial function in situ in permeabilized muscle fibers, tissues and cells.* Nature protocols, 2008. **3**(6): p. 965-76.
9. Votion, D.M., et al., *Physical fitness and mitochondrial respiratory capacity in horse skeletal muscle.* PLoS One, 2012. **7**(4): p. e34890.
10. Norgren, L., et al., *Inter-Society Consensus for the Management of Peripheral Arterial Disease (TASC II).* European journal of vascular and endovascular surgery, 2007. **33 Suppl 1**: p. S1-75.
11. Pipinos, II, et al., *The myopathy of peripheral arterial occlusive disease: part 1. Functional and histomorphological changes and evidence for mitochondrial dysfunction.* Vascular and endovascular surgery, 2007. **41**(6): p. 481-9.
12. Tonkonogi, M. and K. Sahlin, *Physical exercise and mitochondrial function in human skeletal muscle.* Exerc Sport Sci Rev, 2002. **30**(3): p. 129-37.
13. Pesta, D., et al., *Similar qualitative and quantitative changes of mitochondrial respiration following strength and endurance training in normoxia and hypoxia in sedentary humans.* Am J Physiol Regul Integr Comp Physiol, 2011. **301**(4): p. R1078-87.
14. Andreas, M., et al., *Effect of ischemic preconditioning in skeletal muscle measured by functional magnetic resonance imaging and spectroscopy: A randomized crossover trial.* Journal of Cardiovascular Magnetic Resonance, 2011. **13**(1).
15. Little, J.P., et al., *An acute bout of high-intensity interval training increases the nuclear abundance of PGC-1alpha and activates mitochondrial biogenesis in human skeletal muscle.* Am J Physiol Regul Integr Comp Physiol, 2011. **300**(6): p. R1303-10.
16. Hayot, M., et al., *Skeletal muscle microbiopsy: a validation study of a minimally invasive technique.* Eur Respir J, 2005. **25**(3): p. 431-40.

17. Jacobs, R.A. and C. Lundby, *Mitochondria express enhanced quality as well as quantity in association with aerobic fitness across recreationally active individuals up to elite athletes.* J Appl Physiol, 2012.
